# Insect community composition and functional roles along a tropical agricultural production gradient

**DOI:** 10.1007/s11356-018-1818-4

**Published:** 2018-03-30

**Authors:** Angelina Sanderson Bellamy, Ola Svensson, Paul J. van den Brink, Jonas Gunnarsson, Michael Tedengren

**Affiliations:** 1 Sustainable Places Research Institute and School of Planning and Geography, Cardiff University, 33 Park Place, Cardiff, CF10 3BA Wales; 2 Department of Ecology, Environment and Botany, Stockholm University, SE-109 61 Stockholm, Sweden; 3 Department of Aquatic Ecology and Water Quality Management, Wageningen University, Wageningen University and Research Centre, P.O. Box 47, 6700 AA Wageningen, The Netherlands; 4 Alterra, Centre for Water and Climate, Wageningen University and Research Centre, P.O. Box 47, 6700 AA Wageningen, The Netherlands

**Keywords:** Banana production, Management practices, Costa Rica, Insect diversity, Functional roles, Ecosystem services

## Abstract

High intensity agricultural production systems are problematic not only for human health and the surrounding environment, but can threaten the provision of ecosystem services on which farm productivity depends. This research investigates the effects of management practices in Costa Rica on on-farm insect diversity, using three different types of banana farm management systems: high-input conventional system, low-input conventional system, and organic system. Insect sampling was done using pitfall and yellow bowl traps, left for a 24-h period at two locations inside the banana farm, at the edge of the farm, and in adjacent forest. All 39,091 individual insects were classified to family level and then morphospecies. Insect species community composition and diversity were compared using multivariate statistics with ordination analysis and Monte Carlo permutation testing, and revealed that each of the management systems were significantly different from each other for both trap types. Insect diversity decreased as management intensity increased. Reduced insect diversity resulted in fewer functional groups and fewer insect families assuming different functions essential to ecosystem health. Organic farms had similar species composition on the farm compared to adjacent forest sites, whereas species composition increasingly differed between farm and forest sites as management intensity increased. We conclude that while organic production has minimal impact on insect biodiversity, even small reductions in management intensity can have a significantly positive impact on on-farm insect biodiversity and functional roles supported.

## Introduction

Agricultural systems depend on ecosystem services, including but not limited to, pollination, pest control, nutrient cycling, and soil conditioning (Daily 1997). When these services are compromised, it can be costly for producers to replace them (Klein et al. 2007; Losey and Vaughan 2006). Intensive agriculture, with its high inputs of agrochemicals, has been shown to seriously degrade these services (Kremen 2002). Already in the 1980s, concerns were voiced over how biodiversity loss could impact ecosystem functioning (Ehrlich and Ehrlich 1981; Myers 1990;Wilson 1988, 1989). At this time, Schulze and Mooney (1993) presented arguments for the hypothesis that greater diversity could lead to increased productivity, greater efficiency in the use of limiting resources, and increased ecosystem stability. In the late 1990s, Loreau et al. (2003; Yachi and Loreau 1999) theorized through ecological modeling that greater biological diversity (i.e., biodiversity) would lead to greater community stability.

By 2001, there was a consensus that many species are needed to maintain stability of ecosystem functioning, particularly in the face of environmental changes (Loreau et al. 2001). Research shows that higher diversity leads to functional complementarity which increases productivity and nutrient retention; some ecosystem processes are unaffected by initial species loss, due to functional redundancy or relatively weak relationships between those species and their living environment; and sometimes relatively rare species can exert a strong influence on ecosystem functioning (Reich et al. 2012; Tilman et al. 2006; Hooper et al. 2005).

In this paper, we focused on an input-intensive commodity of global significance, bananas, and measured insect diversity along a production gradient: from high-input, to low-input, to organic, to natural forest. Insect diversity was partitioned into functional roles and used as an indicator of the depth of provisioning of on-farm ecosystem services. The functional roles studied include recycling/detrivore, fungivore, predator, herbivorous, scavenger, parasitoids, and ants which fulfill multiple functions simultaneously. Diversity of insects and other arthropods (arachnids and acarina) was chosen as the measure of environmental quality in this study because they comprise 90% of the organismal variability of all species. they dominate the structure of ecosystems (Pimentel et al. 1992), and they perform crucial functions to stabilize ecosystems (Wilson 1987), all of which makes them a good measure for biodiversity evaluation (Duelli et al. 1999). Some services that insects provide are decomposition of organic matter and recycling nutrients, conditioning of soil by channeling and moving material to different soil layers, and predation on pest insects. At a broader, landscape-scale, insects also provide pollination services and are an important source of food for birds and mammals. Of all of the insects that have been identified, less than 1% of those are pests.

Banana production is a significant source of foreign exchange income for many tropical countries. Costa Rica is the world’s second largest exporter of bananas; it is economically dependent on the production and export of bananas owing to the large number of jobs and foreign exchange income that the industry generates. Agricultural exports comprise 33.9% of Costa Rica’s foreign exchange earnings (FAOSTAT 2011), of which bananas are the most valuable export. Banana production, however, is also a significant source of pollution, due to the intensive application of fertilizers and pesticides that are used to maintain high levels of productivity (Bellamy 2013; Hernandez and Witter 1996). The use of pesticides in banana production has been linked to watershed contamination, alterations in aquatic community compositions, and human intoxications (Castillo et al. 2000; Castillo et al. 2006; Wesseling et al. 2010). Therefore, it is imperative to identify alternative management practices that reduce the toxic effects of pesticides on humans and non-target organisms in the farm and surrounding environments, including adjacent waterways.

The existence of alternative management practices does not ensure their adoption by banana producers. An important step in this direction is the ability to show the benefits of such changes to producers, e.g., in the form of increased financial profits that result from improved quality of the provision of ecosystem services. To date, however, there is little known about the effects of varying intensities of production in tropical ecosystems on the ecology of the production system, and more specifically, on insect community composition (Kessler et al. 2009; Tylianakis et al. 2006). The research presented here sets out to evaluate whether practices to reduce management intensity lead to an increased insect biodiversity. We test the following hypotheses: (1) that increased management intensity leads to a higher abundance of some insects but lower overall biodiversity in terms of number of insect families, leading to different insect communities; (2) insect communities on farms with increased management will have lower resilience in terms of redundancy within functional roles; and (3) banana farms have decreased insect diversity compared to adjacent forests.

## Materials and methods

### Study area

Structured interviews were conducted at 39 banana farms across the Atlantic zone in Costa Rica, in order to identify banana farms with different management intensities (Bellamy 2013). From these 39 interviews, 16 banana farms were sampled for this study, based on their level of management intensity.

**Fig. 1:**
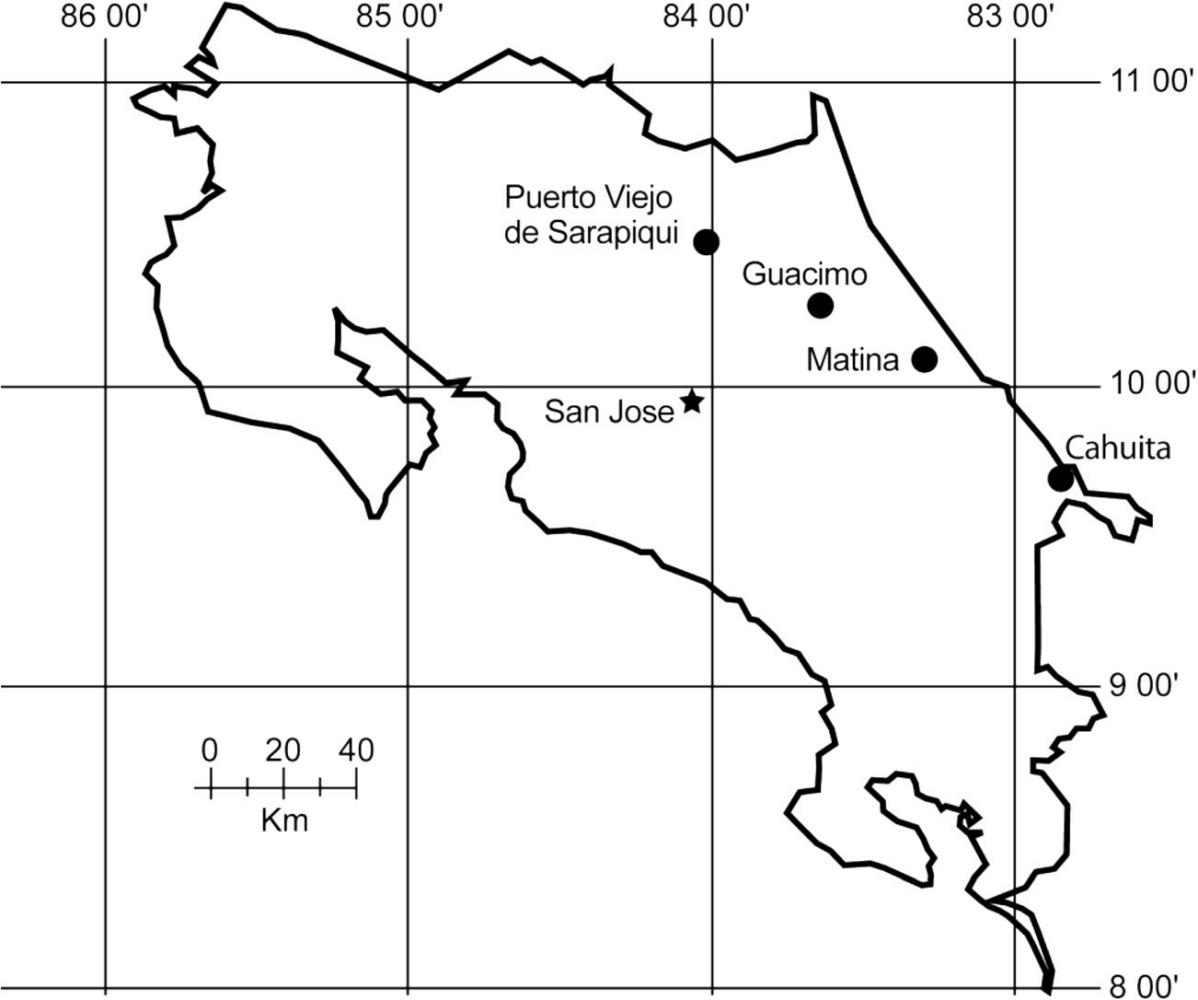
Map of Costa Rica, with the different sampling areas indicated

Nine farms were large-scale high-input conventional monocultures of banana production located in the Atlantic zone: Matina and Puerto Viejo de Sarapiqui (Fig. 1). The farm sizes ranged from 121 to 307 ha (Table 1) and were 8–17 years old. Two farms were large-scale low-input conventional monocultures of banana production owned by Earth University, where managers continuously implement new practices developed by researchers and students with the aim of producing bananas more sustainably. One such practice is the recycling of banana waste into manure that is then applied to the soil on the farm. Both farms received the same regular aerial fungicide application compared to the other conventional producers, but one farm made only one application of nematicide/year, whereas the other farm made two applications/year. The following nematicides were used on banana farms: carbofuran, terbufos, oxamil, ethoprophos, phenamiphos, and cadusafos. Fourteen different fungicides were used on high-input farms: difenoconazole, tridemorph, tebuconazole, bitertanol, pymetrozine, azoxystrobin, tiabendazole, pyraclostrobin, benomil, mancozeb, chlorothalonil, trifloxistrobina, propiconazole, and spiroxamina. The first farm only received organic manure, whereas a mix of organic and synthetic fertilizer was used on the second farm. No herbicides were used on either farms, and these farms instead relied on manually chopping weeds (Table 1); herbicides used on high-input farms were paraquat, diquat, and glyphosate.

**Table 1:** Average values for the three farm management types studied: organic and high-input and low-input farms. *N* is the number of farms in the category. Mean size (ha) is the average size of the farms in hectares. The following three columns refer to the number of herbicide applications, nematicide applications, and fungicide applications, respectively, per year. Insecticide-impregnated bags refer to whether or not farms used them (all high- and low-input farms used them, while none of the organic farms did) to cover the banana bunch during the maturation phase. The final two columns refers to the average number of times manually weeding per year and the average number of other crops cultivated on the farm

Farm type	*N*	Mean size (ha)	No. of herb. apps	No. of nem. apps	No. of fung. apps	Insecticide-impregnated bags	No. of times man weeding	No. of other crops	Fertilizer (kg/ha/year)	Yield (kg/ha/year)
Organic	5	13.6	0	0	0	No	3.8	9.2	155	3840
High input	9	226	7.3	3	54	Yes	1	0	2787	59,020
Low input	2	84	0	1.5	46	Yes	10	.5	1924	40,860

Five farms were small-scale and had received organic certification for their banana production. They varied in size, ranging from 2 to 25 ha, and were between 10 and 20 years old (Table 1). They also varied with regard to the diversity of other crops grown on the farms. More details of the interview survey from the 39 farms are presented elsewhere (Bellamy, 2013).

Climatic conditions in Costa Rica are dictated largely by elevation and which side of the central mountain range sites is located. As all farms were located in the Atlantic coastal zone, they all experienced similar climatic conditions of precipitation, humidity, and temperature (Hall 2000). The elevation range for all 16 farms was from 0 to 126 m above sea level, average annual rainfall varies between 245 and 490 cm, and average temperature is approximately 27 °C. Soil temperature taken at each sampling site varied between 26 and 28 °C.

### Sampling methods

**Fig. 2:**
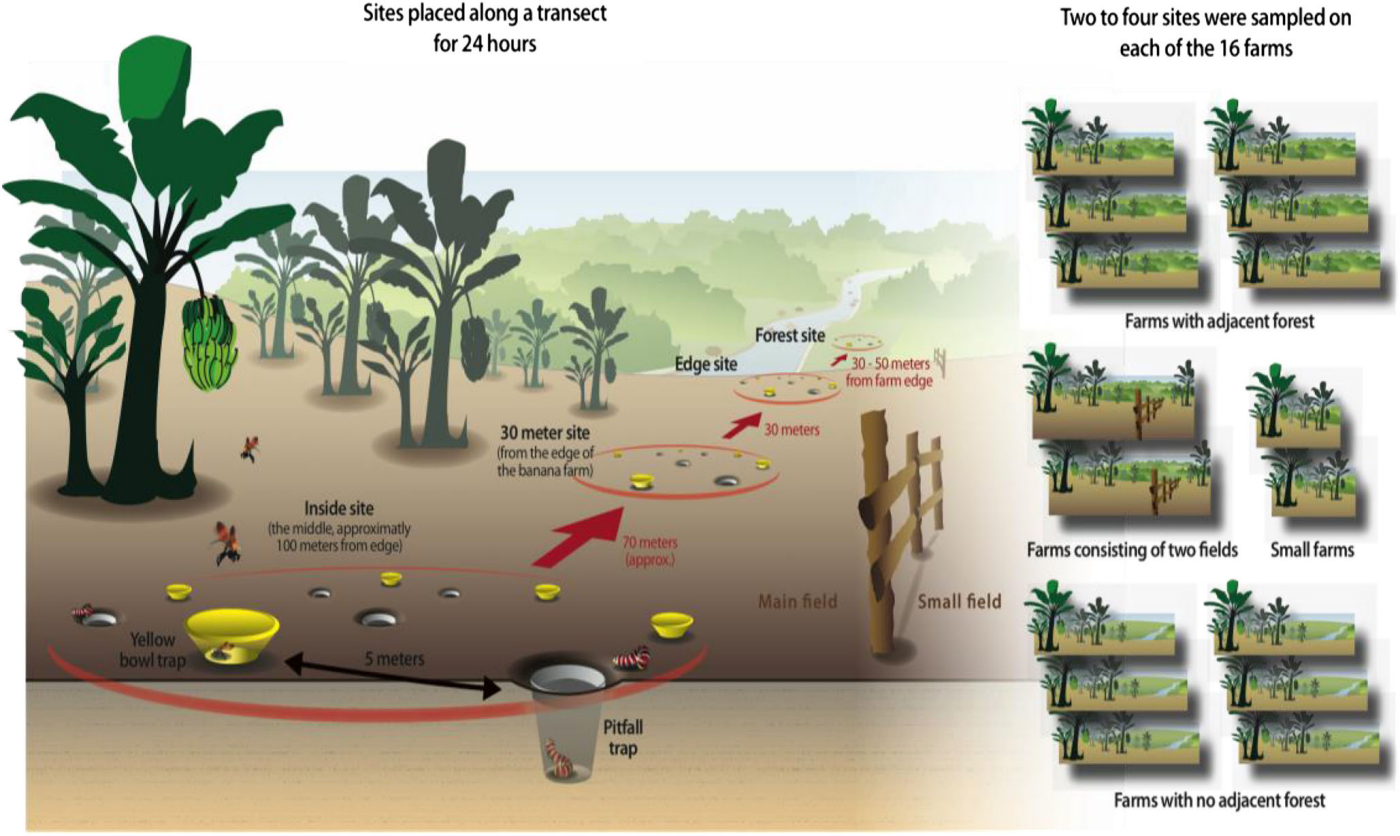
Schematic illustration of sampling design

Insect sampling was conducted using pitfall and yellow bowl traps. Two to four sites were sampled on each of the 16 farms, depending on the size and neighboring habitat of each farm. In all cases but two, farms consisted of one large field site; on the two farms that differed from this pattern, the larger of the two farm fields was chosen for sampling. The sites were placed along a transect running from (1) the middle of the farm, referred to as the inside site; (2) 30 m from the edge of the banana farm; (3) the edge of the banana farm; and (4) in the forest bordering the banana farm (Fig. 2). There were five replicate yellow bowl traps and five replicate pitfall traps at each site. Each trap was placed at least 5 m apart according to Sutherland (2006). Traps were left in place for 24 h. At two small farms, the site that was 30 m from the edge also constituted the inside site. On six farms, there was no adjacent forest, so this point was not sampled in these cases. The specific site for each sampling point was decided in advance after consulting a map of the farm and surrounding land uses. In cases where there was not forest adjacent to the farm, the forest site was not sampled, and the edge site was placed adjacent to rivers (*n* = 3) or open pasture (*n* = 3).

The choice of running a transect from the forest, to the edge, to inside of the farm was to use forest sites as a reference site for area undisturbed by farm management practices; the traps for the forest site were placed 30–50 m from the edge site. The 30 m from the edge site was chosen in order to cancel out the possibility of differing edge effects on farms of different sizes; on a large farm, the middle is further from the edge than on a small farm. Thus, the 30 m from the edge point is a standardized point of comparison between farms. The inside point was approximately 100 m from the edge of the farm. Sampling took place during the Costa Rican dry season, in March 2007.

### Yellow bowl and pitfall traps

Yellow bowl traps are useful for catching flying insects, especially diptera, hymenoptera, hemiptera, and homoptera which are all attracted to the bright yellow color of the traps. The traps were 12-oz, yellow, Solo brand, plastic bowls. Water, pre-mixed with blue Dawn detergent soap (3–4 ml soap/l of water; LeBuhn et al. 2003) was poured into the bowl, approximately 3 cm deep. The soil surface was cleared of debris and the bowl placed on the flat ground.

Pitfall trap types are useful for catching living, surface-dwelling insects such as Coleoptera (beetles) and Formicidae (ants). Hard plastic bowls with straight sides, 15 cm deep, and a circumference of 44 cm were dug into the ground so that the lip of the bowl was even with ground level. The same water-soap mixture was used as for the yellow bowl traps, and was poured into the bowl, approximately 3 cm deep. A plastic lid cover, propped up approximately 3 cm above the lip of the bowl with the use of three popsicle sticks/lid, was used to keep rain water and debris from falling into the bowl (Sutherland, 2006).

In the lab, the mixture for both the pitfall traps and the yellow bowl traps were sieved through a 0.5-mm mesh net, rinsed, and then preserved in 70% alcohol solution until taxonomic identification. Each sample was labeled and kept separate for identification. Identification was conducted to family and then morphospecies, using a stereoscope. One specimen of each morphospecies from each sampling location was preserved in alcohol and deposited at the National Institute of Biodiversity (Instituto Nacional de biodiversidad, INBio) in Costa Rica for their own species database which records the locations where species are captured.

### Data analysis

While traditional descriptors such as species richness, species abundance, and diversity indices are often used to measure anthropogenic impact on natural communities, they fail to relate any information about changes in community composition that may significantly influence ecosystem functioning and the provision of essential ecosystem services (Kremen 2005; Tscharntke et al. 2005; Tylianakis et al. 2007). Thus, we chose to study the effect of management type and sampling location on insect species diversity with multivariate statistics, using two ordination techniques: principle component analysis (PCA) and redundancy analysis (RDA). Multivariate analyses are used frequently in ecotoxicology to describe differences in community composition among sites and to relate these differences to a chemical/management treatment (see for instance Kedwards et al. 1999; Van den Brink et al. 2003). While PCA selects the linear combination of species that gives the smallest total residual sum of squares, RDA also considers the linear combination of explanatory variables in order to analyze how well the explanatory variables explains the species data (Ter Braak 1995); thus, we primarily used RDA.

PCA and RDA analyses generate ordination diagrams that allow one to compare how closely the different sites are related to each other in terms of species composition and how the species composition varies between treatments, i.e., management types. Sites that lie close together on the diagram share a more similar species composition than those sites that lie further apart (Ter Braak 1995). Species points lying far away from the center of the diagram are important for indicating sample differences; the further away, the larger the difference. Those species whose abundance shows variation from the mean are displayed in the diagram. In addition to the visual representation of data provided by these ordination techniques, the statistical significance of hypothesized differences was obtained using Monte Carlo permutation testing according to Ter Braak and Šmilauer (2002).

To test the first hypotheses, we performed a RDA analysis using species data from the inside and the 30-m sampling sites on each farm. Nominal variables denoting the management type of the samples were introduced as explanatory variables, while nominal variables denoting whether samples were taken at the inside and 30-m sites were introduced as covariables in the analysis.

In order to test the second hypothesis, we charted the functions performed by each insect family in the RDA analysis used to test the first and second hypotheses. Insects were assigned to different functions based on exploitation of the same resource in a similar way, while ants form a functional guild of their own, owing to the diverse number of functions they perform (Moran and Southwood 1982; Ugalde 2002; Zumbado 2006). If the family was represented in the RDA analysis for either pitfall or yellow bowl trap, it was included once, and assigned based on which management type it was most strongly associated.

To test the third hypothesis, we performed a RDA for the pitfall traps and yellow bowl traps data set. These analyses were performed for each farm management type separately, using sampling location (i.e., inside, 30 m, edge, and forest) as explanatory variables and farm as covariable. Monte Carlo permutation tests were run to evaluate the differences in species composition between sample locations for each management type separately.

## Results

In all of the traps combined, we captured 38,091 individual insects, representing 239 families—67% in pitfall traps and 33% in yellow bowl traps. The following 19 orders were represented: Anoplura, Coleoptera, Collembola, Dermaptera, Diptera, Hemiptera, Homoptera, Hymenoptera, Isoptera, Lepidoptera, Neuroptera, Odonata, Orthoptera, Plecoptera, Psocoptera, Thysanoptera, Thysanura, Trichoptera, and Zoraptera. A summary of the collected insect data is presented in Table 2. Results show that on average, the high-input farms had the fewest number of insects trapped per farm; approximately 7% more were captured on organic farms and 14% more on low-input farms. As the intensity of management practices decreased, the average number of morphospecies caught increased. Across all farm types, more species were trapped at the edge sites compared to the other sampling sites. There were fewer insects caught at the forest sites compared to the other sites for the low-input and organic farms, but the high-input farms had more insects caught in the forest sites compared to the inside sites. When comparing insects caught in forest sites adjacent to organic farms with insects caught in forest sites adjacent to both high- and low-input farms, 86% of the families found in forest adjacent to organic farms were also found in forests adjacent to conventional farms.

**Table 2:** Summary of insect data. All data is presented as the average number of individuals caught per site for both yellow bowl and pitfall traps combined. The second column is the average value of insects caught per site for all farm management types (e.g., for the 16 farms sampled, on average 2795 individual insects were trapped), whereas the last three columns present the average values per farm management type

Average no. of:	Overall	High input	Low input	Organic
Morphospecies/farm	208	193	214	234
Arthropods found on the farm	2795	2745	3133	2946
Arthropods found at the inside site	677	618	791	826
Arthropods found at the 30-m site	714	609	778	878
Arthropods found at the edge site	867	815	1031	897
Arthropods found at the forest site	557	703	534	346
Coleoptera individuals	64 (2.2%)	43	42	112
Collembola individuals	1473 (53.5%)	1533	1834	1300
Diptera individuals	226 (8.0%)	170	187	362
Hemiptera individuals	10 (.4%)	5	16	18
Homoptera individuals	155 (5.6%)	148	194	170
Hymenoptera individuals	671 (24.1%)	589	835	788
Orthoptera individuals	33 (1.2%)	37	38	28
Acarina individuals	47 (1.7%)	50	36	47
Araneida individuals	67 (2.5%)	76	77	51

**Fig. 3:**
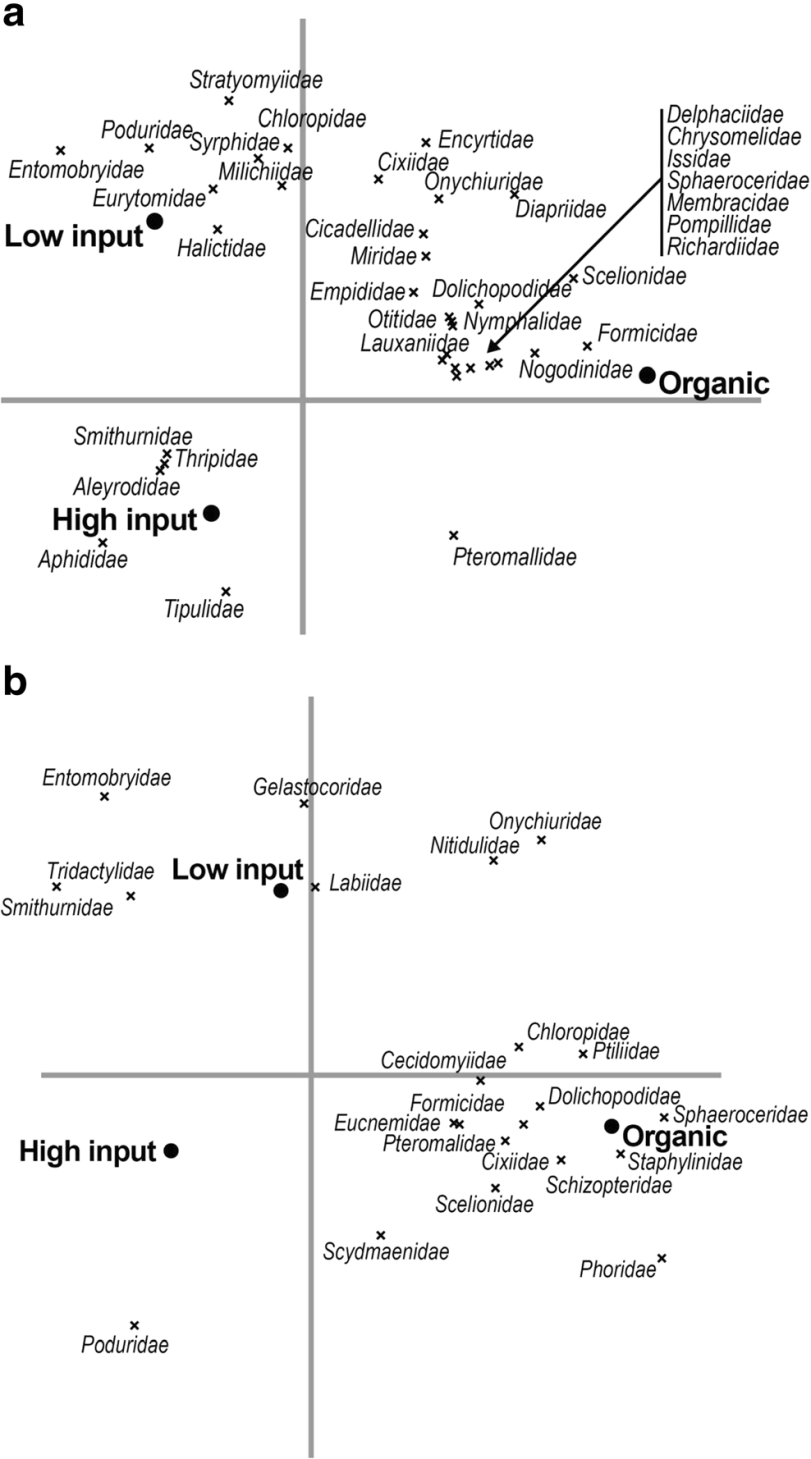
**a** Yellow bowl trap RDA biplot showing the differences in species composition between management styles using only the inside and 30-m samples. The inside and 30-m samples were introduced as covariables, which explained 2% of the variation in species composition. Management style explained 10% of which 73% is displayed on the horizontal axis and another 27% on the vertical axis. **b** Pitfall trap RDA biplot showing the differences in species composition between management styles using only the inside and 30-m samples. The inside and 30-m samples were introduced as covariables, which explained 1% of the variation in species composition. Management style explained 10% of which 67% is displayed on the horizontal axis and another 33% on the vertical axis

We tested our first hypothesis that increased management intensity leads to greater abundance of some insects but lower overall biodiversity (Fig. 3). Results of the Monte Carlo permutation tests evaluating the differences in species composition between management styles separately gave significant *p* values for all combinations (*p* ≤ 0.002), meaning that the species community composition sampled in the organic and low- and high-input farm groups was all significantly different from each other for both trap types.

The RDA biplots in Fig. 3 show that most of the species cluster to the right side of the axis, together with the organic management type. There are fewer species clustered close to the low-input site and there are very few species that lie in the same quadrant as the high-input site. Furthermore, each of the points representing different management styles lie quite far from each other and in different quadrants, indicating that their species community composition are distinctly different from each other. In Fig. 3b, for example, one can see that the abundance of the family Poduridae has a strong positive association with farms using high-input management practices and a negative one with low-input and organic farms.

To further test our first hypothesis, the yellow bowl and pitfall trap biplots in Fig. 3 both show that each of the three management styles has invertebrate community assemblages that differ from each other; this is represented in the biplots by site points that lie far away from each other and confirmed by the Monte Carlo permutation tests (*p* ≤ 0.002). The first axis shows the difference between the organic farms on one hand, and the high- and low-input farms on the other hand; these differences in species composition are therefore the largest. The second axis differentiates the organic and the low-input management types, herewith indicating smaller differences between these management types.

**Fig. 4:**
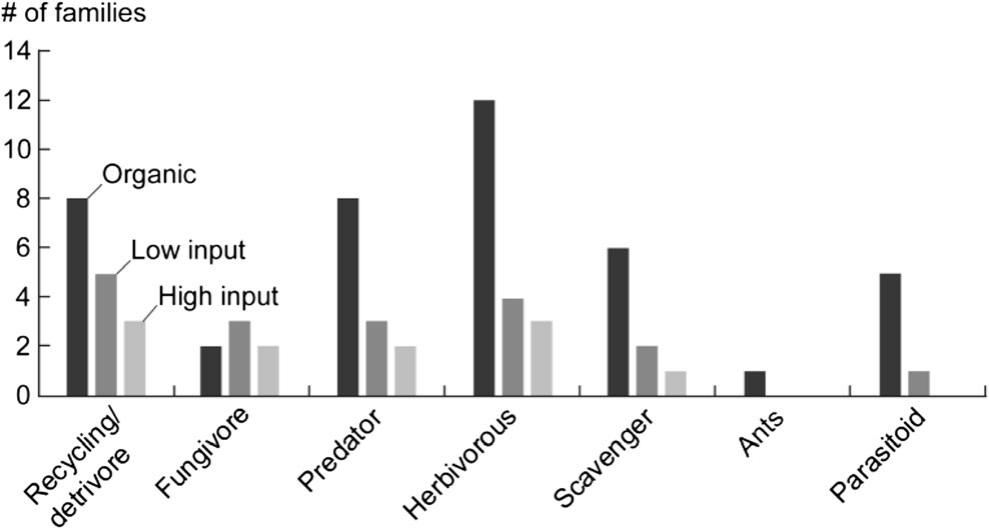
Functional roles represented by insect families positively correlated with organic and low- and high-input farms for both pitfall and yellow bowl traps, as displayed in Fig. 3

To test our second hypothesis, Fig. 4 shows how the positive association of different families with the three different management regimes relates to the representation of different insect functions (see Appendix. Table 4 for list of families and their assigned functional role). The organic farms are positively associated with a larger number of families performing each functional role compared to low- and high-input farms, with the exception of fungivores. Neither the low- nor the high-input farms are positively associated with ants, and the high-inputs farms also lack a positive association with any parasitoids.

**Fig. 5:**
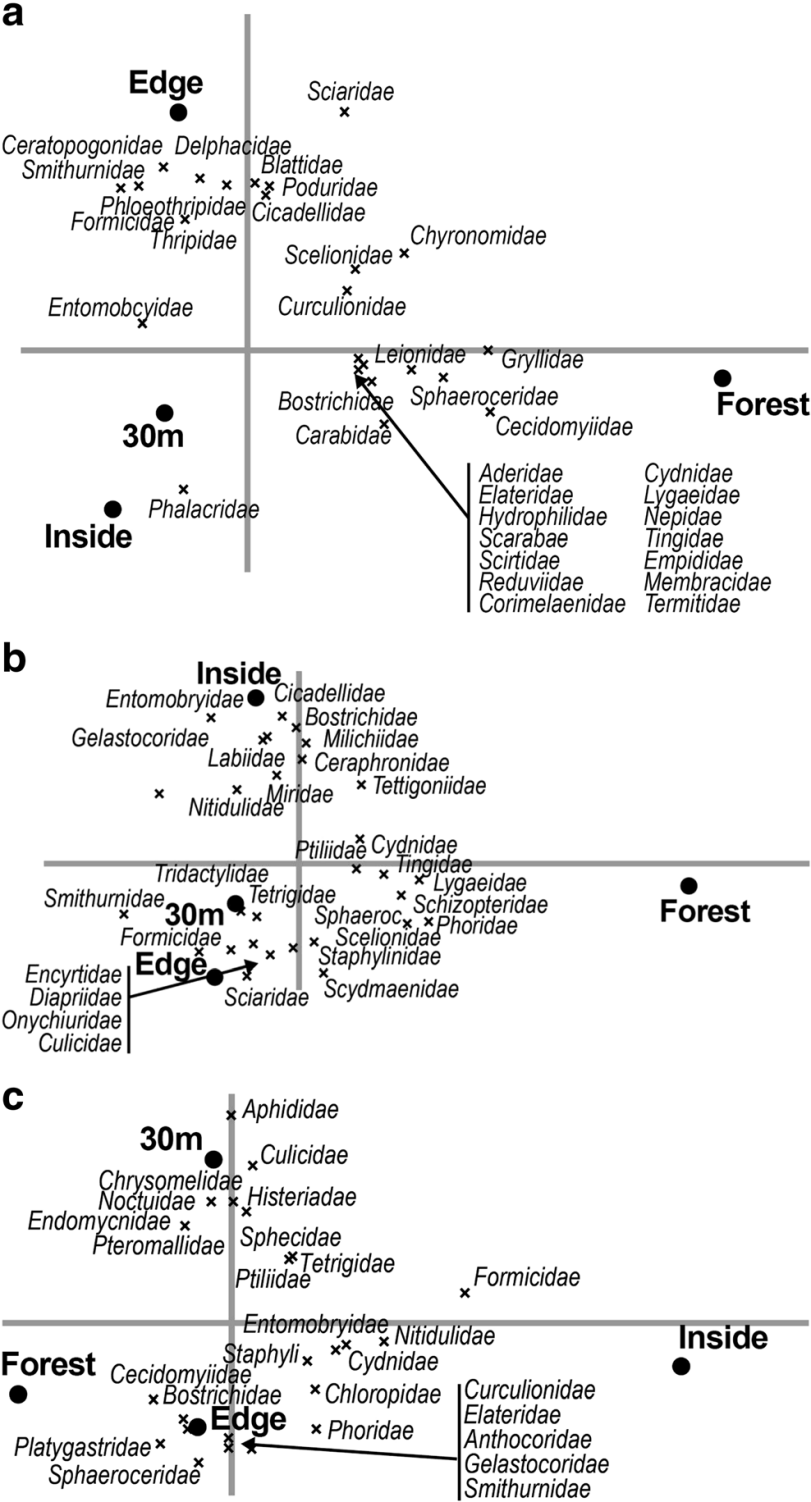
**a** Pitfall trap RDA biplot showing the differences in species composition between sampling locations at the high-input farms. Farm was introduced as covariable, which explained 28% of the variation in species composition. Sample location explained 6% of which 64% is displayed on the horizontal axis and another 31% on the vertical axis. **b** Pitfall trap RDA biplot showing the differences in species composition between sampling locations at the low-input farms. Farm was introduced as covariable, which explained 28% of the variation in species composition. Sample location explained 11% of which 63% is displayed on the horizontal axis and another 26% on the vertical axis. **c** Pitfall trap RDA biplot showing the differences in species composition between sampling locations at the organic farms. Farm was introduced as covariable, which explained 38% of the variation in species composition. Sample location explained 5% of which 62% is displayed on the horizontal axis and another 23% on the vertical axis

Our third hypothesis was that banana farms have a lower invertebrate diversity compared to neighboring forests (Fig. 5; Table 3).

**Table 3:** Pitfall trap analyses showing *P* values given from Monte Carlo permutation tests evaluating the differences in species composition between site locations for each management separately

	Organic	High input	Low input
Inside vs 30 m	0.002	0.544	0.001
Inside vs edge	0.001	0.001	0.001
Inside vs forest	0.001	0.001	0.001
30 m vs edge	0.320	0.002	0.066
30 m vs forest	0.320	0.001	0.001
Edge vs forest	0.280	0.001	0.001

For the pitfall traps, we see that species community composition at the forest sampling sites is significantly different from the inside, 30 m, and edge sampling sites, with the exception of the organic farms for the 30 m and edge sampling sites (Table 3). The RDA biplots show an increasing difference between sites with regard to the number of families represented as management intensity increases. For the high-input management style, most species are placed to the right of the y-axis where the forest site point is also located, with a gradient from high species diversity to low diversity moving from the forest site to the edge, 30 m, and then inside points (Fig. 5a). The low-input farms also have the forest site on the right side and the inside, 30 m, and edge sites on the left side of the axis, but with an almost equal distribution of insect families on either side of the axis (Fig. 5b). Figure 5c shows that the forest site for the organic farms is located to the left of the y-axis together with the edge and 30-m sites, while the inside site is to the right, where more of the species also lie. *P* values in Table 3 also show that the inside site for the organic farms is significantly different from the other three sites, but that the forest, edge, and 30-m sites are not statistically different from each other. Many of the species in this biplot lie along the y-axis, displaying neither a strong relationship to the inside or the forest site. Analysis of yellow bowl traps yielded very similar results. These results illustrate that the difference in species composition between the inside and forest sites on organic farms is caused by a few species only (e.g., Formicidae). From the analyses, it can thus be concluded that while high- and low-input farms with neighboring forest patches have decreased insect diversity compared to their neighboring forests, the diversity in the organic farms on the other hand is similar to the adjacent forest.

## Discussion

As Table 2 and Fig. 3 illustrates, our first hypothesis was verified, namely that increased management intensity leads to a greater abundance of some insects but to a lower overall insect diversity. Decreases in management intensity do make a difference in insect diversity, as seen in the difference between low- and high-input farms in Fig. 3. Figure 4 shows how this increased diversity translates to a greater number of families performing different functions. Thus, as management intensity increases, there are fewer positive associations with families performing different functions important to ecosystem processes. Different species respond differently to stressors, so redundancy within functional roles can act as an insurance and increase the likelihood that net community function will be maintained after a disturbance or extreme event (Loreau et al. 2003; MEA 2005; Yachi and Loreau 1999). It has been shown that a large pool of species is especially important in intensive agricultural landscapes to maintain ecosystem functioning (Loreau et al. 2001; Tscharntke et al. 2005). Enhanced ecosystem resilience is an important benefit provided by biodiversity (Schmid et al. 2009 and references therein). Loss of biodiversity undermines the resilience of the system, making it more susceptible to sudden environmental changes or management mistakes, and can translate into lower yields for producers, or even system collapse (Daily 1997; Russell 1989).

Insects assume many important functional roles, several of which are important to the banana production system itself. Some insects act as pests and feed on different parts of the banana plant; in the case of bananas produced in Costa Rica, the principal pests are black sigatoka, an air-borne fungus, and the burrowing nematode *Radopholus similis*, but there are other pests, like the virus disease bunchy top (vectored by the banana aphid *Pentalonia nigronervosa*), and *Cosmopolites sordidus* and *Chaetanaphothrips* spp, which are both insects that affect farms sporadically (Ortiz Vega et al. 1999). These different pest species are the target of the heavy pesticide applications used by conventional banana farms. However, as shown in this research, there are on average over 200 other morphospecies of insects found on the farm, that are also adversely affected by the application of pesticides. These non-target organisms may fill functional roles that can be beneficial to the production system. Among these functional roles are predation (by both predators and parasitoids), organic matter decomposition and nutrient recycling, regulation of soil microorganisms, and soil conditioning (soil channeling and moving material to different soil layers).

Aphids, thrips, and whiteflies are all commonly considered pests, and can cause significant economic losses in commercial crops. All three of these families cause crop losses through their feeding on plant material, and in the case of aphids, one particularly important species of aphid, the *Pentalonia nigronervosa* is a disease vector for the bunchy top banana virus. One predator of aphids is the Coccinellidae family of beetles (common name ladybirds/ladybugs); this family of beetles is not positively associated with high-input farms, but both aphids and ladybirds were found in abundance in yellow bowl traps taken from inside and 30 m sites on low-input farms. In general, it has been found that a greater number of predator species acts to suppress aphid populations (Snyder et al. 2006). Thus, a reduction in management intensity can help to positively impact natural predator-pest dynamics. Thrips are also pests to banana production. There are two families, Eulophidae and Trichogrammatidae, that are known to parasitize thrips; other biocontrol agents of thrips include aphid wasps (Van Driesche et al. 2007). Eulophidae species were in high abundance in both the pitfall traps (Fig. 4a) and the yellow bowl traps taken from the forest sites adjacent to high-input farms. This is an important example of how preservation of forest areas within the landscape matrix and the resulting movement of insects across different landscapes can contribute to on-farm pest control.

As mentioned earlier, the organic farms are located in the southern Atlantic zone of Costa Rica, whereas, the high- and low-input farms are located approximately 150 km further north within the Atlantic zone (Fig. 2). It may be possible that some of the differences we see in the data can be attributed to different geographic locations. However, as mentioned in the results, we compared insects found in forest adjacent organic farms with insects found in forest adjacent high- and low-input farms in order to see how insect communities in these two locations differed in undisturbed areas. We found that 86% of families present in forest adjacent organic farms were also present in forest adjacent high- or low-input farms. The presence of many of the same insect families is likely the result of the similar climatic conditions and elevation on all of the farms.

In Fig. 3, the results illustrate that banana farms with different management styles have different insect communities, which was the second hypothesis that we tested. There are more orders of insects represented at the organic farm sites with more families present compared to both the low- and high-input farms (see also Table 2). Furthermore, the insects that are present at the high-input farms tend towards the role of pests. Without the balance in predator-prey relations that comes with a more complex community structure, such as exists on the organic farms (Fig. 4), pest insects become more abundant, which is what is observed on the high-input farms. Parasitoids play a crucial role in biological control of other insects that is central to community structure and function (Hawkins and Sheehan 1994) and are often used successfully in biological control programs; they are usually the most efficient enemy for controlling insect pests (Van Driesche et al. 2007). There are no parasitoid families positively correlated with high-input farms, whereas there are five different parasitoid families positively correlated with organic farms.

Our third hypothesis that banana farms have decreased insect diversity compared to neighboring forest sites was also shown to be true for high- and low-input farms, but not organic farms (Fig. 5). The forests adjacent organic farms have similar community compositions compared to the inside sampling points. This is most likely because the organic management systems sampled in this study are an agroforestry system that closely mimics the forest system; they have a high diversity of overstory tree species, with many understory tree and plant species intermixed throughout the farm. In addition, there is an absence of the use of chemicals, which disrupt the balance between different insect species. Management practices on the low- and high-input farms reduced the diversity of species found on these farms compared to their forest sites. Again, the gradient of management practices does make a difference, as the low-input farms had more diversity of insects at the inside site compared to the inside site on high-input farms.

As shown in Table 1, the high-input farms apply fungicides at a weekly rate, nematicides three times a year, and herbicides an average of 7.3 times per year, whereas the low-input farms, while applying fungicides similarly, applied nematicides an average of 1.5 times per year and abstained from using herbicides. The organic farms abstained from the use of any pesticides. The toxicity value of the specific pesticides used on the farms in this study were calculated (see Kovach et al. 2011 for more details), and the results show that the average toxicity value for the nematicides used in this study are more than twice those for fungicides and herbicides.. While herbicides are not as toxic, they may act to reduce the amount of food plant and habitat structure for beneficial insects. Additionally, the different nematicides used are broad spectrum pesticides, meaning that they are non-selective and affect both pest and beneficial insect species. The largest difference in management practices between the low- and high-input farms is their use of herbicides and nematicides, which may account for the differences that we see in insect community compositions. Organic farms do not apply any pesticides, but they also have a more complex and diverse habitat structure at both the farm-level (they incorporate a number of fruit trees and woody tree species in the canopy level) and the small-scale (increased ground cover with minimal manual weeding), which can contribute significantly to diversity of insect species (Langellotto and Denno 2004).

## Conclusions

The results of this study show that the greater the intensity of management practices in banana production, the greater the effect in reducing diversity of insect populations on banana farms. This can have deleterious effects on production systems, as important functions filled by different insect families are compromised. However, as research in this area still remains largely theoretical, there is a need for research that can quantify the relationship between insect biodiversity and provisioning of ecosystem services, for example the extent to which biodiversity may be lost before it affects the provision of ecosystem services.

It is critical that less intensive practices for banana production—such as increasing diversity in on-farm tree species, understory ground cover, and overstory canopy species (all of which leads to increasing structural habitat complexity), which can in turn reduce the amount of pesticides needed, both of which may have positive effects on insect species community composition—be utilized. This benefits not only the health of the surrounding environment and communities, but also the long-term health of the production system that relies on services provided by a diverse and thriving insect community. It is encouraging that our results show that even small differences in management intensity can have a positive impact on community composition, and that agroforestry systems can support community compositions that closely mimic those found in forest. In an era of increasing expansion of intensive agricultural production systems, more attention is warranted for the benefits of low-input extensive agricultural systems, especially in tropical systems, where this has historically been the norm and still forms an important part of the landscape.

## Appendix

**Table 4:** List of families used for functional role analysis, the functional role assigned and the reference

Aleyrodidae	Sap/disease vector	Kaufman, 2007
Anthocoridae	Predator	Borror and White, 1970
Aphididae	Sap	Borror and White, 1970
Bostrichidae	Lignivorous	Solis, 2002
Braconidae	Parasitoid	Ugalde, 2002
Cantharidae	Pollen/nectar/predator	Solis, 2002
Cecidomyiidae	Herbivorous/pollinator/predator	Zumbado, 2006
Ceratopogonidae	Pollen/nectar/detrivore	Zumbado, 2006
Chloropidae	Herbivorous/predator/detrivore/parasitoids	Zumbado, 2006
Chrysididae	Parasitoid	Ugalde, 2002
Chrysomelidae	Herbivorous	Solis, 2002
Cicadellidae	Sap	Borror and White, 1970
Cixiidae	Herbivorous	Bourgoin et al. 2004
Cydnidae	Sap	Dolling, 1981
Coccinellidae	Predator	Solis, 2002
Culicidae	Nectar/pollen	Zumbado, 2006
Curculionidae	Herbivorous	Solis, 2002
Delphacidae	Herbivorous	Cook and Denno, 1994
Diapriidae	Parasitoid	Ugalde, 2002
Dolichopodidae	Predator	Zumbado, 2006
Drosophilidae	Detrivore/parasite	Zumbado, 2006
Dryinidae	Parasitoid	Ugalde, 2002
Elateridae	Predator/herbivorous	Solis, 2002
Empididae	Nectar/predator	Zumbado, 2006
Encyrtidae	Parasitoid	Ugalde, 2002
Endomychidae	Fungivore	Borror and White, 1970
Entomobryidae	Detrivore	Hopkin, 1997
Ephydridae	Scavenger/nectar	Zumbado, 2006
Eucnemidae	Detrivore/xylophages	Borror and White, 1970
Eurytomidae	Parasitoid/herbivorous	Ugalde, 2002
Evaniidae	Predator	Ugalde, 2002
Evaniidae	Parasite	Borror and White, 1970
Formicidae	Ants	Ugalde, 2002
Gelastocoridae	Predator	Borror and White, 1970
Halictidae	Parasite/pollen	Borror and White, 1970
Histeridae	Predator	Solis, 2002
Ichneumonidae	Parasitoid	Ugalde, 2002
Issidae	Herbivorous	Wilson et al., 2004
Labiidae	Scavenger	Burton, 2001
Lauxaniidae	Detrivore	Zumbado, 2006
Lygaeidae	Seed/sap	Borror and White, 1970
Membracidae	Sap	Borror and White, 1970
Milichiidae	Scavenger	Borror and White, 1970
Miridae	Sap	Borror and White, 1970
Mordellidae	Pollen/nectar/detrivore	Solis, 2002
Muscidae	Detrivore/nectar/pollen/predator	Zumbado, 2006
Mymaridae	Parasitoid	Ugalde, 2002
Nitidulidae	Detrivore	Solis, 2002
Noctuidae	Herbivorous	Borror and White, 1970
Nymphalidae	Herbivorous/nectarivores	Borror and White, 1970
Onychiuridae	Detrivore	Hopkin, 1997
Otitidae	Herbivorous/detrivore	Borror and White, 1970
Phlaeothripidae	Fungivore	Moritz et al., 2001
Phoridae	Scavenger/predator/parasites/parasitoid	Zumbado, 2006
Piophilidae	Scavenger	Borror and White, 1970
Platygastridae	Parasitoid	Ugalde, 2002
Poduridae	Detrivore	Hopkin, 1997
Pompilidae	Parasitoid	Ugalde, 2002
Pteromalidae	Parasitoid	Ugalde, 2002
Ptiliidae	Detrivore/fungivore	Borror and White, 1970
Reduviidae	Predator	Borror and White, 1970
Richardiidae	Detrivore	Zumbado, 2006
Scelionidae	Parasitoid	Ugalde, 2002
Schizopteridae	Predator	Slater and Baranowski, 1978
Sciaridae	Detrivore/nectar	Zumbado, 2006
Scydmaenidae	Predator	Borror and White, 1970
Sminthuridae	Detrivore	Hopkin 1997
Sphaeroceridae	Detrivore	Zumbado, 2006
Sphecidae	Predator	Ugalde, 2002
Staphylinidae	Predator	Solis, 2002
Stratiomyidae	Detrivore/nectar	Zumbado, 2006
Syrphidae	Pollinator/detrivore/predator	Zumbado, 2006
Tachinidae	Pollinator/parasitoid	Zumbado, 2006
Tetrigidae	Herbivorous	Borror and White, 1970
Thripidae	Herbivorous/pollinator	Borror and White, 1970
Tipulidae	Nectar/detrivore	Zumbado, 2006
Tridactylidae	Detrivore/herbivore	Borror and White, 1970
